# Microstructure of the juvenile sheep aortic valve hinge region and the trilamellar sliding hypothesis

**DOI:** 10.21542/gcsp.2020.23

**Published:** 2020-11-30

**Authors:** Magdi Yacoub, Yuan-Tsan Tseng, Brian Mitchelson, Najma Latif

**Affiliations:** 1 NHLI, Imperial College, Heart Science Centre, Harefield Hospital, Harefield, Middx. UB9 6JH; 2 Qatar Research Foundation, Doha, Qatar

## Abstract

**Background:** The aortic valve mechanism performs extremely sophisticated functions which depend on the microstructure of its component parts. The hinge mechanism of the aortic leaflets plays a crucial part in the overall function. However, the detailed microstructure and its relation to function has not been adequately studied.

**Methods:** The aortic roots of juvenile sheep were fixed under physiologic pressure. Sections through all three sinuses were then performed to illustrate the microstructure of the hinge mechanism in different regions of the aortic root.

**Results:** The hinge region in the different sinuses showed unique microstructure with a trilamellar topology with a dominant core consisting of glycosaminoglycans. The exact arrangement of the trilamellar structures varies around the aortic sinuses, which could have functional implications. These features allow the hinge to perform its complex functions through what we have described as “the trilamellar sliding hypothesis”.

**Conclusion:** The microstructure of the hinge mechanism is unique and enables it to perform it sophisticated functions.

## Introduction

The aortic valve and root perform extremely sophisticated functions which depend on the macro- and microstructure of its component parts^1,2^. These components include the annulus, the sinuses, the sinotubular junction and importantly the leaflets. Each leaflet includes 3 components; a “belly”, a co-apting surface, and a hinge. The specialised unique macro- and microstructures of each of these parts is responsible for the ability of the leaflets to move smoothly and rapidly from the open to the closed position and back during each cardiac cycle^1,3–6^. In addition, the hinge plays a major part in optimising the shape and dynamism of the effective aortic orifice, thus enhancing ventriculoaortic coupling^7,8^.

Here we show in a series of images, the detailed microstructure of the hinge, which we believe plays a crucial part in the kinematics of the aortic leaflets. The study was performed in juvenile sheep to represent young mammalian hearts, free of age-related degeneration or disease.

## Material and Methods

The protocol for the study was approved by the Qatar Foundation ethics committee. Five juvenile sheep aortic roots were examined (6 months old).

### Sample preparation

All valves were excised and washed in phosphate buffered saline. These were fixed in 10% formal saline for 24 hours. Radial sections were cut from each valve from the base of attachment to the free co-apting edge through the centre of the leaflets and along the sinus walls. These were then embedded in paraffin. They were all processed at the same time for conventional histological analyses using 5 μm thick sections after de-waxing in xylene.

### Alcian blue/Picrosius Red staining

These were performed as previously published^9,10^. Tissue sections were dewaxed and hydrated with water. Sections were stained with Weigerts haematoxylin (equal volume of 1% haemotoxylin in methanol and 1.2% w/v ferric chloride, 1% v/v conc. hydrochloric acid) for 10 mins. They were rinsed briefly in running water and differentiated in acid alcohol followed by washing with water until clear. Alcian Blue (0.5%w/v Alcian Blue 8GX and 1% v/v glacial acetic acid) was applied for 10 mins and rinsed briefly in distilled water. Molybdophosphoric acid (1%w/v) was applied for 20 mins followed by rinsing briefly in distilled water. Sirius Red / picric acid (1% w/v Sirius Red in saturated picric acid) was applied for 60 minutes and rinsed briefly in distilled water. The sections were dehydrated and mounted in DPX.

## Results

In all aortic sinuses, the hinge consisted of a triangular structure, with its base partially or completely embedded in the aortic annulus and its apex continuing into the leaflet. The triangle consists of:

**Figure 1.**
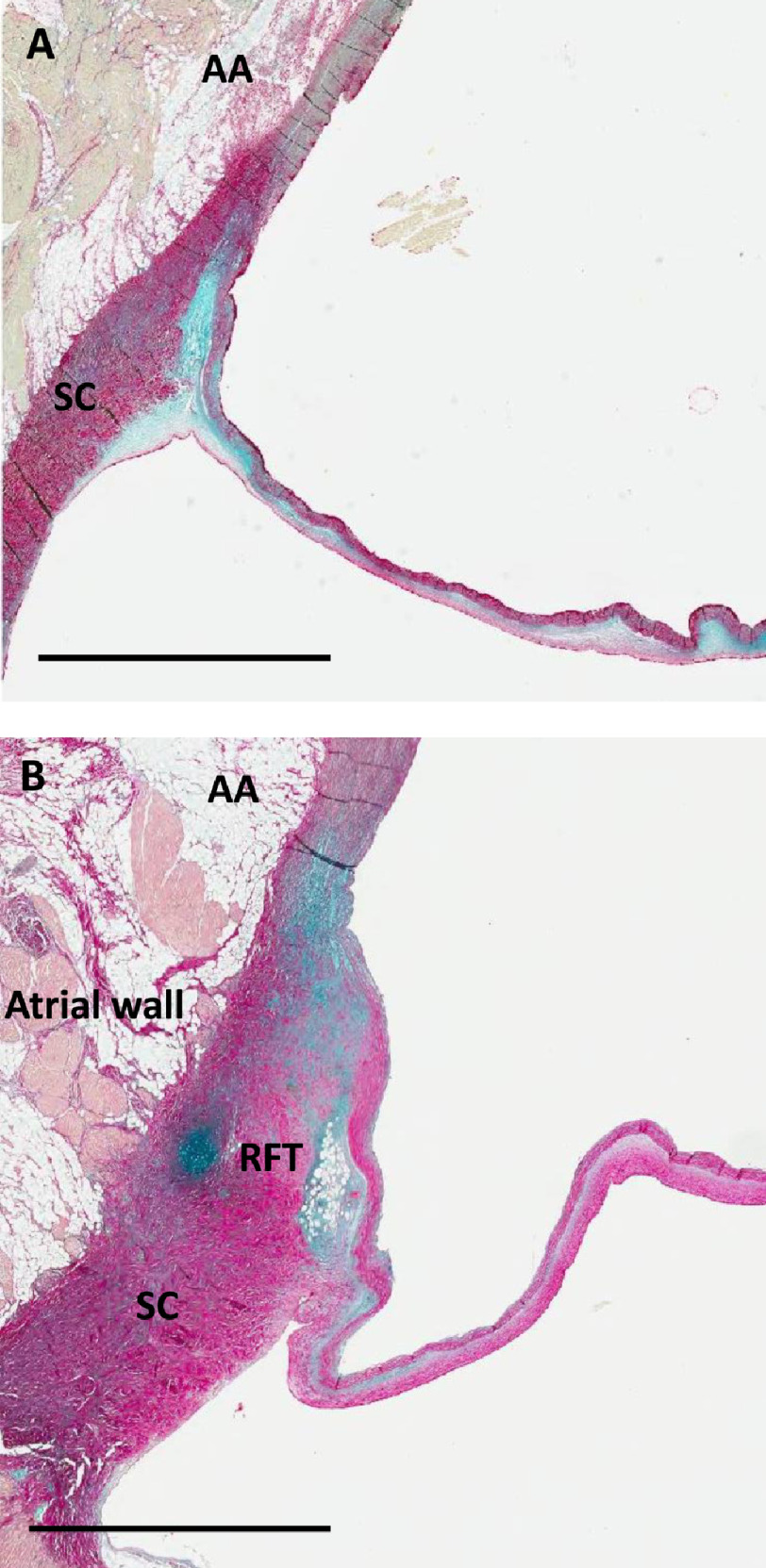
Section through the posterior half of the non-coronary sinus showing aorto-atrial space and attachment of the hinge mechanism to the annulus, subaortic curtain and aortic wall and aortic media (A) and non-coronary leaflet attached to the middle of the non-coronary sinus showing the attachment of the hinge to the right fibrous trigone (B). AA, aorto-atrial space; SC, subaortic curtain; RFT, right fibrous trigone. Scale bar is 3 mm.

**Figure 2.**
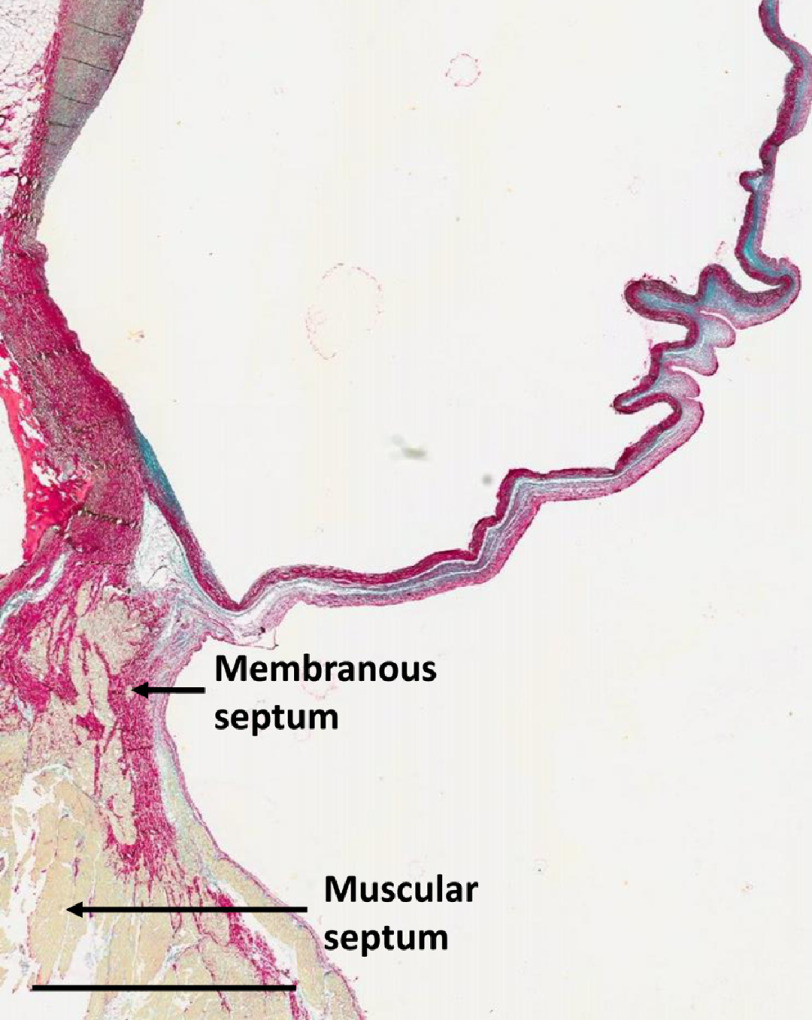
Section through the anterior half of the non-coronary sinus approaching the interventricular septum. Scale bar is 3 mm.

**Figure 3.**
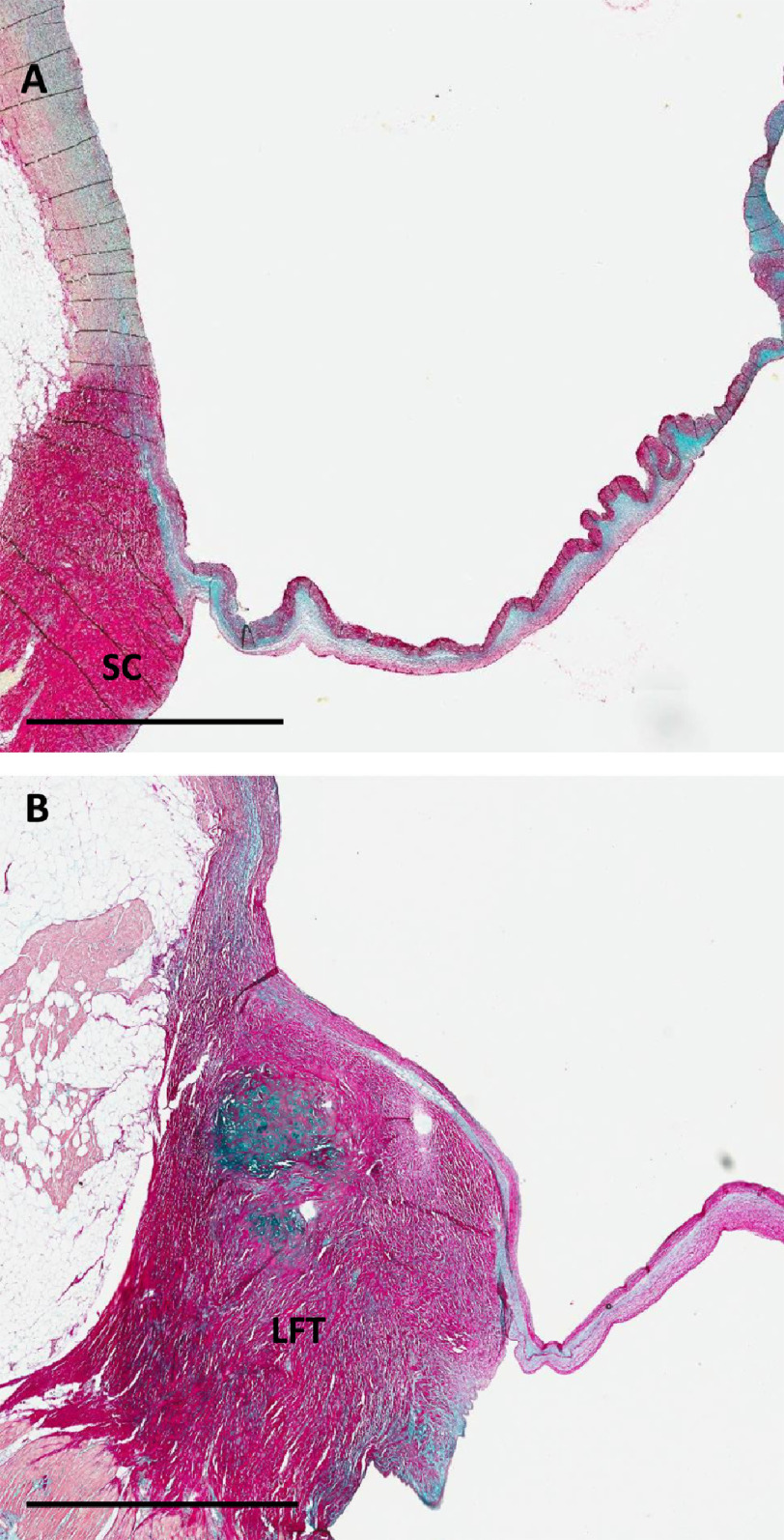
Section through the anterior part (A) and through the posterior part (B) of left coronary sinus showing the left fibrous trigone (LFT). SC, subaortic curtain. Scale bar is 3 mm.

**Figure 4.**
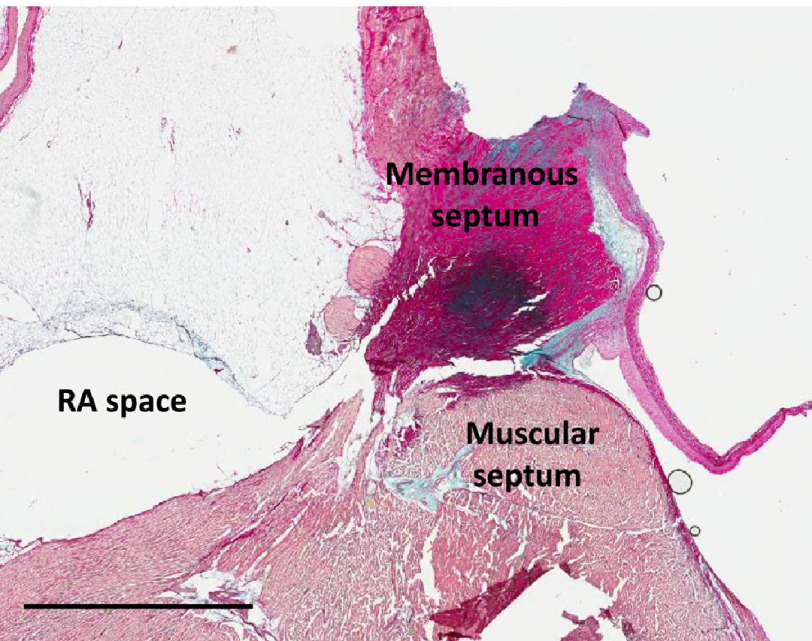
Section through the anterior part of right coronary sinus showing the right coronary leaflet sitting on the septum. Scale bar is 3 mm.

• A central core, occupying approximately 70 per cent of the hinge area, and formed mainly of glycosaminoglycans (GAGs) (Figures 1–4).
• A fibrosa, which is located on the arterial side of the core and consists of parallel sheets of collagen, linking the media of the aorta and throughout the aortic annulus and the fibrosa of the leaflet. This ensures continuity of the longitudinal curvature of the aortic sinus into the leaflet (Figures 1–4), and
• the ventricularis (elastica) which is located on the ventricular aspect of the hinge, consisting of elastic tissue, and which links the LV endocardium to the elastica of the valve (Figures 1–4).

The transverse section of the hinge, and closely related annulus, varied in size and shape in the three sinuses - particularly in the right coronary sinus - due to the presence of the “muscle bar” (Figure 4). In this region, the cross section of the annulus consisted of two parts, a circular fibrous body which provided robust fixation to the media of the aortic sinus, and a thinner flat extension on the surface of the ventricular muscular septum. The hinge structure was attached to the ventricular flat extension of the annulus (Figure 4).

## Discussion

This study serves to show the detailed microstructure and topology of the mammalian aortic hinge region. The hinge has a specific trilamellar microstructure, with a dominant core consisting of glycosoaminaglycans. This component provides the annulus with the capacity to bend as well as change its size and shape during different parts of the cardiac cycle (dynamism) and thus reduce energy loss during systolic ejection of blood, allowing the outflow tract to act as a smooth surfaced tube. The fibrosa of the hinge provides the capacity of the hinge to suspend the leaflet, while maintaining the combined curvature of the aortic sinus and leaflet. The elastica allows the hinge to regain its shape, following deformation during the different phases of the cardiac cycle.

***The Trilamellar Sliding hypothesis: The microstructure and topology of the hinge strongly suggests that the hinge achieves its varied functions by sliding of the three layers over each other, aided by the fluid-like GAGs in the core.***

### Limitations

This study was performed in a small number of juvenile sheep. However, the observations were very clear and should provide the basis for similar studies in different mammalian species, furthermore, we have observed similar findings in humans (not shown).

## Conclusions

The structure-function relationship described in this study could have important implications to understanding the complex functions of the living aortic valve mechanism and importantly in tissue engineering.
